# Development of new levodopa treatment strategies in Parkinson’s disease—from bedside to bench to bedside

**DOI:** 10.1080/03009734.2017.1285374

**Published:** 2017-03-03

**Authors:** Sten-Magnus Aquilonius, Dag Nyholm

**Affiliations:** Department of Neuroscience, Neurology, Uppsala University, Uppsala, Sweden

**Keywords:** Carbidopa, drug development, intestinal gel, levodopa, microtablets, Parkinson’s disease

## Abstract

This review will illustrate the process of moving from an idea through preclinical research and Galenic developments into clinical investigations and finally to approval by regulatory agencies within the European Union. The two new treatment strategies described, levodopa/carbidopa intestinal gel and levodopa/carbidopa microtablets, for advanced Parkinson’s disease, have been developed in collaborative research within departments at Uppsala University. With this historical approach, reference priority is given to reports considered to be of special importance for this more than two decades long process ‘from bedside to bench to bedside’.

## Background

Historically, anticholinergic drugs, introduced with scopolamine by Ordenstein at Salpêtrière in Paris, was the sole pharmacotherapy for Parkinson’s disease (PD) for about 100 years (1). The Russian-born pathologist Trétiakoff, also working in Paris, observed nigral degeneration as a hallmark of PD about 45 years before the nigro-neostriatal dopamine pathway was mapped by Swedish scientists (2). In this group Arvid Carlsson, Nobel laureate in 2000, made the pivotal discovery of DA as a neurotransmitter (3). During this golden age of Swedish neuroscience (4) important ground-breaking observations were also reported from Oleh Hornykiewicz and collaborators in Vienna. The profound depletion of striatal DA in advanced PD was found (5). Further, the Austrian scientists pioneered dopaminergic therapy showing a marked but short-lasting reduction in parkinsonian akinesia after intravenous administration of dihydroxyphenylalanine (DOPA) (6). It took another 6–8 years before George Cotzias ascertained that slowly increased dosing of oral DOPA via its active enantiomer L-DOPA (levodopa) dramatically reduces the cardinal features of PD (7,8). Introduction of tablets with levodopa combined with an inhibitor of levodopa decarboxylation, with no penetrance to the CNS, is the last milestone during these years of pioneering research and represents the appearance of development and marketing by the ‘big pharma’ industries (9). Some important milestones in the history of knowledge concerning the pathophysiology and treatment of PD are summarized in Table 1.

**Table 1.** Milestones in Parkinson’s disease research.

Year	Milestone	Authors (ref.)	Country
1817	An essay on the shaking palsy	Parkinson J. (61)	UK
1868	Treatment with scopolamine	Ordenstein L. (1)	France
1919	Degeneration of substantia nigra	Trétiakoff C. (2)	France
1958	DA neurotransmitter	Carlsson A. et al. (3)	Sweden
1960	DA deficit in PD brain	Ehringer H. and Hornykiewicz O. (5)	Austria
1961	Therapeutic effect of i.v. DOPA	Birkmayer W. and Hornykiewicz O. (6)	Austria
1964	Mapping of nigro-striatal DA neurons	Anden N.E. et al. (4)	Sweden
1967	Oral DOPA therapy	Cotzias G.C. et al. (7)	USA
1969	L-DOPA introduced	Cotzias G.C. et al. (8)	USA
1971	L-DOPA/decarboxylase inhibitor	Pletscher A. and Bartholini G. (9)	Switzerland

## Progress and limitations

Animal experiments have demonstrated a dopaminergic inhibition on striatal cholinergic interneurons (10). The therapeutic effect of centrally active anticholinergic agents in PD is commonly explained by their ability to compensate for the effects of cholinergic ‘overactivity’ due to dopamine depletion.

While levodopa treatment offered therapeutic effects far superior to the anticholinergic agents, pioneering clinicians working on transforming the PD treatment reported several side-effects. Data from an early study with levodopa by Melvin Yahr and collaborators from 1969 depicted the abundant side-effects at that time, with nausea, involuntary movements, and vomiting affecting a majority of the patients (11). Without a decarboxylase inhibitor, only a small proportion of orally administered levodopa is transported into the brain by means of the large neutral amino-acid transporter system and decarboxylated into dopamine at the site of action within the striatum. The main part of levodopa administered without a decarboxylase inhibitor will then be metabolized to dopamine in peripheral organs inducing ‘peripheral side-effects’. Circulating dopamine stimulating receptors within the area postrema, localized outside the ‘blood–brain barrier’ (BBB), triggers nausea, the most common side-effect. Other side-effects were due to dopamine interaction with cardiovascular receptors at different peripheral sites. However, in these early studies also central side-effects, in particular involuntary movements, choreatic dyskinesias, were common and troublesome especially in patients with the most advanced disease.

Following the introduction of peripherally active decarboxylase inhibitors, benserazide (Hoffman la Roche) and carbidopa (Sandoz), peripheral side-effects markedly decreased and the effective dose of levodopa could be reduced by about 75%. However, dyskinesias and fluctuating symptoms came even more to the fore.

## Early studies on fluctuations in symptomatology and the hypothesis of continuous dopaminergic stimulation

As mentioned above, pioneering clinicians noticed the relationship between motor fluctuations and dyskinesia and the severity of PD. These treatment problems were never noticed in newly diagnosed PD patients, and the term ‘honeymoon period’ was introduced for this initial stage. Later on, the relationship between levodopa dosage and mobility became successively obvious with variation between mobility (‘on’), akinesia (‘off’), and periods with dyskinesia—choreatic movements related to ‘on’ or dystonic movements mainly related to ‘off’ (12). These clinical observations of dose-related variations in symptomatology formed the starting-point for research on the pharmacokinetics and pharmacodynamics of levodopa treatment in patients at different stages of PD. In an eye-opening study in 1975, Shoulson and collaborators at NIH infused levodopa intravenously to five severely ‘on–off’ fluctuating patients. At constant plasma levodopa concentrations fluctuations virtually disappeared (13). In the following years the ability to prevent and reduce motor fluctuations by constant intravenous levodopa administration was verified by several PD research groups in the UK (14) and in the USA (15). In a pivotal investigation the NIH group registered the reappearance of parkinsonian symptomatology after sudden withdrawal of a constant intravenous optimal dose infusion of levodopa to 48 patients at different stages of PD (16). Patients with the most advanced disease, with ‘on–off’ fluctuations, deteriorated very rapidly, i.e. exhibited a rapid ‘wearing-off’ of levodopa effect. Newly diagnosed patients retained the therapeutic effect, while in stable patients and patients with moderate tendency to fluctuations the symptoms reappeared gradually. The authors introduced the idea of ‘reduced buffering capacity’ as a pedagogic interpretation of the decreasing ability to store dopamine formed due to denervation of striatal dopaminergic nerve terminals.

In 1986, preliminary observations by Kurlan and collaborators indicated that continuous intraduodenal infusion of a levodopa solution can reduce on–off fluctuations as effectively as intravenous delivery (17). Confirmed in several studies, this report opened up an interest in developing a delivery system for continuous availability of levodopa at the site of active absorption in the upper segment of the small intestine (18).

## Investigations of levodopa pharmacokinetics and pharmacodynamics in Uppsala

**Figure 1.**
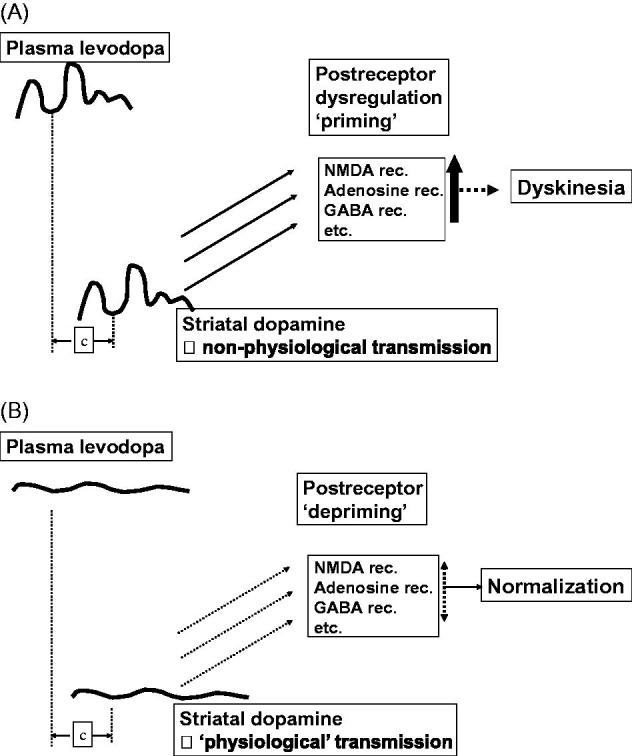
Theoretical sketches of the relationship between mode of delivery of dopaminergic drugs and the development of motor complications including dyskinesias. A: The non-physiological stimulation of dopamine receptors with pulsatile drug delivery, priming for dyskinesias; several other neurotransmitter systems, including inhibitory GABA and excitatory glutamate signalling, are involved in this complex process. B: A suggested primary treatment strategy where continuous drug delivery provides more physiological stimulation of dopamine receptors, a ‘depriming’, and a subsequent normalization of motor fluctuations and dyskinesias.

The introduction, at Uppsala PET-Centre, of novel ^11^C-labelled tracers for pre- and postsynaptic sites in the brain (19–22) made functional investigations of the striatal dopaminergic system possible in different stages of PD (23,24). It could be demonstrated by simultaneous measurement of clinical effects and cerebral kinetics that even in very advanced stages of PD the striatal ability to decarboxylate exogenous levodopa is preserved and a motor response is obtained at low concentrations of striatal dopamine formed (25). Not all symptoms are levodopa-responsive in late-stage PD, but the typical cardinal symptoms still respond (26). The cerebral decarboxylase activity may decline with time but is obviously well preserved for many years (27). Furthermore, the close time-effect relationship between the precursor, levodopa in plasma, and striatal synaptic dopamine in advanced PD could be indirectly shown using displacement of the dopamine D2-receptor antagonist ^11^C-raclopride (28). High-resolution pharmacokinetic/effect studies in PD patients revealed that variation in plasma levodopa concentration is the determining factor for motor fluctuations also in patients on clinically optimized combination therapy with dopamine agonists and enzyme inhibitors (29). Based on the afore-mentioned information, an oversimplified sketch of the pathophysiology of motor fluctuations and dyskinesia was drawn and a primary treatment strategy suggested (Figure 1).

## Development of a levodopa/carbidopa intestinal gel (LCIG)

By means of video scoring, frequent optoelectronic evaluation of motor performance, and plasma levodopa analysis we could depict the pharmacokinetic/-dynamic relationship in advanced PD in great detail. The advantage of stable plasma levodopa levels after intravenous as well as intestinal infusion, via a nasoduodenal route, was confirmed and an interindividual variation in optimal steady state concentrations of plasma levodopa from about 0.3 to 3.0 μg/mL was demonstrated (30). The results encouraged us to develop a clinically acceptable system for continuous duodenal delivery of levodopa/carbidopa to enable individually fine-tuned stable plasma concentrations in advanced PD. With this goal a start-up company, NeoPharma, with Uppsala University as one share-holder, was founded in 1994.

For obvious reasons long-term intravenous delivery of levodopa is not a practical treatment option. Intraduodenal infusions offered a more plausible alternative, and several short-term studies—and some over periods of several months—were reported during the period 1988 to 1998 (see 2004 review by Nyholm and Aquilonius (18)). However, although these early studies confirmed the effectiveness of ‘continuous dopaminergic stimulation’ (CDS) in advanced PD, they were troublesome from different reasons: (1) A naso-duodenal tube is intolerable for long-term use. (2) Water solutions were used, and due to the limited solubility of levodopa in water the patients had to carry large volumes of infusate. (3) The chemical stability of levodopa and carbidopa is low, and frequent renewal of the solution is necessary.

**Figure 2.**
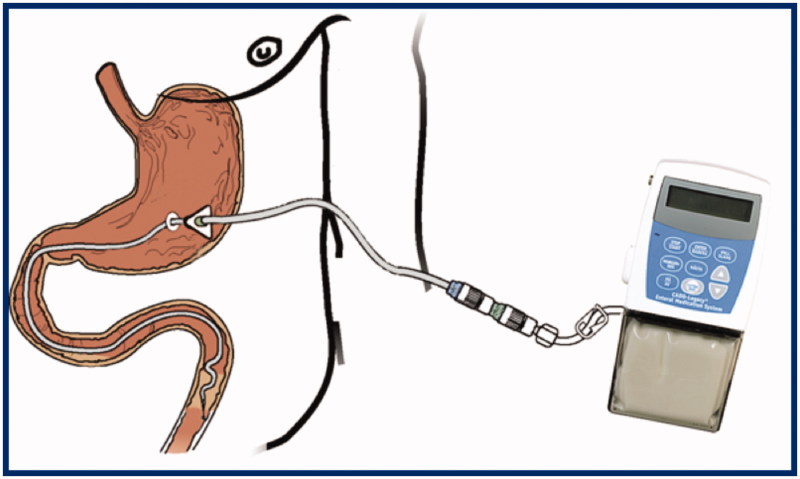
Infusion system for levodopa/carbidopa intestinal gel.

In collaborative research and development between neurology and galenic pharmacy at Uppsala University a new levodopa/carbidopa formulation, a pseudoplastic gel, for infusion by a portable pump via a transabdominal port (Figure 2) was invented and evaluated in a series of clinical investigations (31,32). The aqueous suspension contains 20 mg/mL of micronized levodopa and 5 mg/mL of carbidopa in a methylcellulose gel contained within a cassette of 100 mL. Chemical and physical stability is high, and one cassette normally covers the demand for one day (i.e. 2000 mg of levodopa). The tubing system usually consists of a percutaneous endoscopic gastrostomy tube with a duodenal/jejunal extension tube inside. Dislocation of the intestinal tube was common in the early tube systems but improved with time (33).

The clinical effects of this levodopa/carbidopa intestinal gel (LCIG) were investigated in several comparative studies. In a randomized cross-over trial of LCIG versus oral sustained release tablets over 3 weeks for each treatment, LCIG significantly increased ‘on’-time and decreased ‘off’-time periods and dyskinesias. These positive effects were related to a decrease in the average intra-individual variation coefficient (CV) for plasma levodopa concentrations (34). In a long-term follow-up of 28 patients with very advanced PD treated with LCIG via a transabdominal port, positive effects were sustained in several patients for 4–7 years (35). Further, LCIG monotherapy was shown to be clinically superior to a number of individually optimized combinations of conventional drugs in patients with motor fluctuations (36). Observational data on long-term 24-hour infusion of LCIG has been reported (37).

The LCIG, named Duodopa^®^, was approved by the Swedish Medical Products Agency (MPA) for therapy of very advanced PD with motor fluctuations and dyskinesia in 2004, closely followed by European Medical Agency (EMA) for the rest of Europe. In the following years the positive effects of LCIG were verified in a number of open trials of different format, and also non-motor symptoms and quality of life seemed to improve (38,39). In the USA a large controlled investigation, using a ‘double-blind, double-dummy’ technique, was performed, with a positive outcome, before the approval of ‘Duopa^®^’ by the FDA in 2015 (40), i.e. 20 years after the project started within NeoPharma.

Uppsala-based NeoPharma was sold to the Belgian company Solvay in 2006, and the LCIG product is presently owned and marketed world-wide by AbbVie. Large-scale studies investigating safety and effect on health-related quality of life and non-motor symptoms have recently confirmed the pioneering data and contributed to further understanding (41,42). LCIG provides stable striatal dopamine availability as recently demonstrated in a study using serial PET investigations (43), by means of ^11^C-raclopride displacement (28).

## On the development of levodopa/carbidopa microtablets and an electronic dose dispenser

Early in the development of the LCIG treatment it became obvious that individually fine-tuned dosing is as important as continuous delivery for effective levodopa therapy in advanced PD (44). Could the concept of CDS and individualization be important already in earlier stages of the disease, and could invasive therapy be postponed by improved oral formulations? It is known from rodent and primate models of PD that pulsatile stimulation by dopaminergic drugs has a priming effect for the development of dyskinesia (45). This might imply that the mode of drug delivery in earlier stages of PD is important. Different technologies for sustained release formulations of levodopa tablets have been developed in the past and at present, but as a general principle slow absorption and/or incomplete bioavailability makes individualized fine-tuned dosing hard to achieve.

Under fasting conditions, peripheral pharmacokinetics following oral levodopa is similar in healthy controls and in PD patients at different stages of the disease (46,47). Accordingly, and in line with basic pharmacokinetics, frequent individualized dosing of a rapidly soluble drug with short plasma half-life (for levodopa about 1.5 hour) would be the ideal strategy for oral formulations to obtain optimal stability of plasma concentrations (48). Available levodopa/carbidopa and levodopa/benserazide tablets with levodopa content of 50, 100 and 200 mg are far from ideal for this treatment strategy.

**Figure 3.**
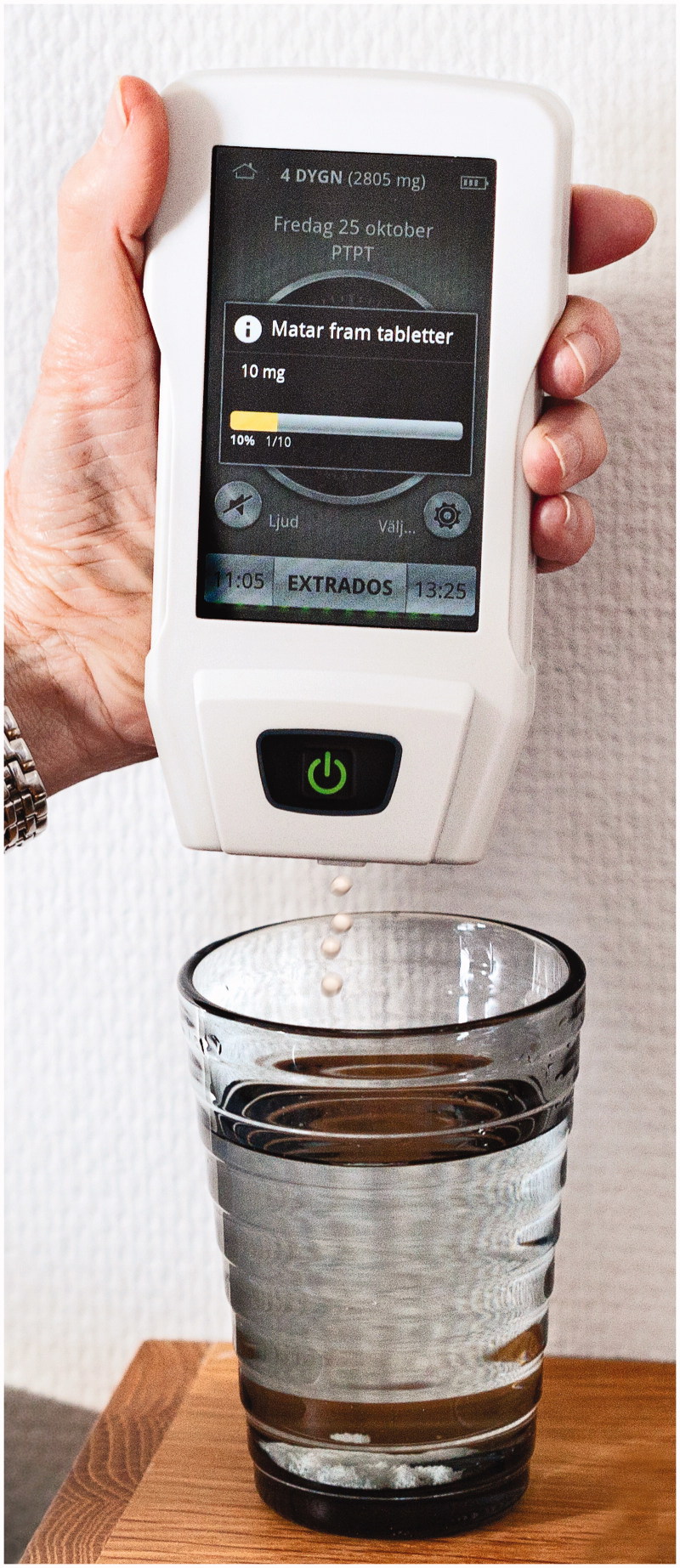
Automatic dose dispenser for levodopa/carbidopa microtablets.

A rapidly soluble tablet formulation containing 5 mg levodopa and 1.25 mg carbidopa was developed (LC-5, named Flexilev^®^ in Sweden). To handle these small tablets with a diameter of 3 mm, in the following referred to as microtablets LC-5, a dosing device is required. This automatic dose dispenser was developed in co-operation with an advisory board of people with PD, and prototypes were evaluated by patients in different stages of disease (49). The final version (Figure 3), CE-classified as a medical device in 2014 and named MyFID (My Flexible Individual Dosing), provides a number of functions. A cassette, containing 750 microtablets (3750 mg levodopa, thus roughly a week’s supply) is docked into the dispenser. A basal individual dosing programme with, for example, at least six intakes per day is chosen by the physician. The schedule is presented on the touch screen, and the patient is reminded of dosing by an alarm. Motor and non-motor symptoms can be reported into an electronic diary (Supplemental Figure, available online) and presented at the next visit to doctor or nurse who in collaboration with the patient can further fine-tune the levodopa delivery if needed. Information on adherence, in addition to need for and reason for extra dosing, is also registered. A group of experts from several European drug approval agencies recently referred to the Flexilev/MyFID concept as one incentive to ensure better pharmacotherapy for older people (50).

The microtablet, LC-5, was approved by the Swedish MPA in 2014. Two pharmacokinetic studies were fundamental for the approval. The drug bioequivalence following oral dosing of the micro-tablets is similar to conventional levodopa/carbidopa tablets allowing a direct transition of treatment (51). Further, in a cross-over study in healthy volunteers, fractionation of levodopa with Flexilev as compared to standard administration of levodopa/carbidopa/entacapone decreased the fluctuation index of both levodopa and carbidopa in plasma by nearly half (52). We have recently confirmed the similarity in levodopa pharmacokinetics in healthy volunteers and PD patients under fasting conditions (53). Based on this knowledge and the importance of CDS and individualized dosing, it can be concluded that the LC-5/MyFID strategy might offer the optimal administration of oral levodopa available today for PD patients with motor fluctuations.

From 2016, LC-5 is reimbursed in Sweden for treatment of patients with advanced PD, and EMA approval has recently been granted for 13 other European countries.

## Ongoing research and development

After the clinical introduction and general acceptance of LCIG therapy in advanced PD, several follow-up investigations and experience-based reports have been published, but the level of evidence is still limited, partly due to the complexity in designing double-blind studies with LCIG (54–56). Technical improvements of the infusion system have also been in focus, and a transcutaneous port has been described as an alternative to conventional PEG-J tubes (57–59).

For individualized fine-tuned dosing with levodopa/carbidopa, valid information on symptomatology is crucial. Based on positive experience of real-time capture of motor function by means of an electronic diary (60), instruments for objective symptom quantification may become part of future versions of the dose dispenser (61). The dispenser offers unlimited possibilities for data storage.

The development of LCIG has served as a proof of concept that individualized, continuously administered levodopa doses are beneficial for patients with fluctuating PD symptoms. Based on this, in parallel with the microtablet concept, a number of new levodopa delivery systems are under development, for example oral sustained-release or gastro-retentive formulations, subcutaneous levodopa infusion, and intestinal levodopa/entacapone/carbidopa infusion (62,63).

## Conclusions

To conclude, it is encouraging that ideas born when meeting patients in a challenging clinical situation have been developed via academic research into marketed medicinal products. Individualization of levodopa therapy is important for people with PD, and further improvement can be foreseen.

## Supplementary Material

Supplemental data
