# Supplementary material for: Development of new levodopa treatment strategies in Parkinson’s disease—from bedside to bench to bedside
Source: Ups J Med Sci. 2017 Mar 3;122(2):71–7. doi: 10.1080/03009734.2017.1285374 (PMC5441375; doi:10.1080/03009734.2017.1285374)
Supplement: Supplemental data [file IUPS_A_1285374_SM2311.pdf]

MyFID behandlingsrapport

Dosintag

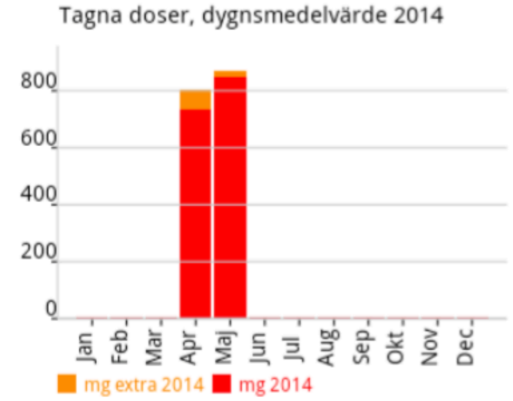

Följsamhet

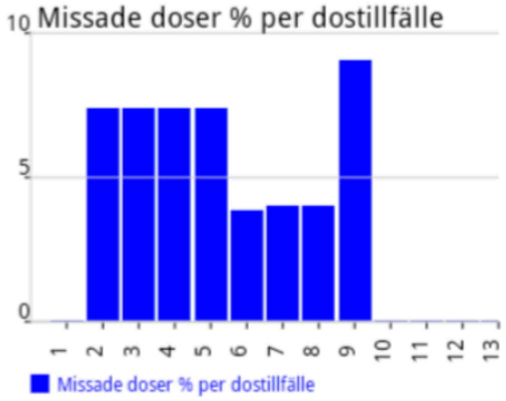

Behandlingsordination Flexilev

Morgondos:	110 mg	(+20 mg, -20 mg)
Dagsdos:	8 st	
	100 mg	(+20 mg, -20 mg)
Extrados:	50 mg	(+20 mg, -20 mg)
Dygnsdos:	910 mg	

Behandlingsutfall:

senaste 2 mån (33 dagar)

Morgondos:	Medel (mg)	115.0
Dagsdos:	Medel antal doser	5.8
	Medeldos (mg)	97.0
Extrados:	Medel antal doser	0.0
	Medel (mg)	44.0
Dygnsdos:	Medel (mg)	708.0

Missade doser:

Totalt för perioden	13
Medel per månad	6.5

Symptom och rörlighet

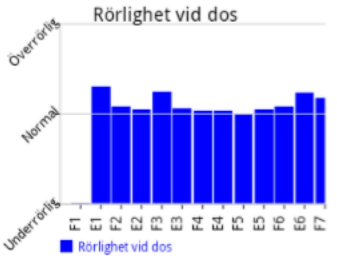

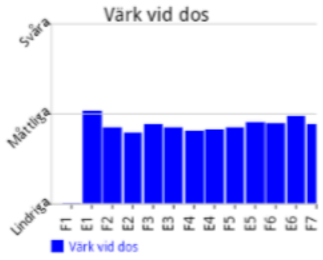

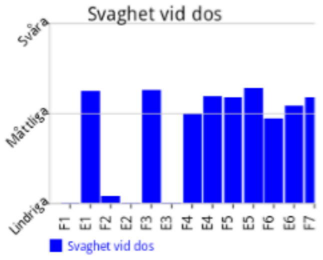

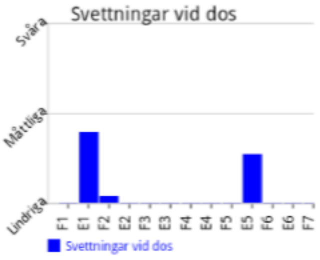

Statistik

Antal ordinerade symptominmatningar: 217

Antal inmatade symptom: 455

Antal extra inmatade symptom: 8

Utskriftsdatum: 2014-05-12 14:46
